# Internet Health Care Service Use Behavioral Pattern Among Older Adults and the Role of the Technology Acceptance and Social Ecological Theory Model: Cross-Sectional Survey

**DOI:** 10.2196/78037

**Published:** 2026-01-15

**Authors:** Rui Li, Xinyu Xu, Qingsong Li, Haobiao Liu, Ting Ting Zhou, Abebe Feyissa Amhare, Peiyu Liu, Jing Tang, Wei Wang, Fuju Zheng, Jing Han

**Affiliations:** 1 Shandong Center for Disease Control and Prevention, Ji'nan, Shandong, China; 2 Key Laboratory of Environment and Genes Related to Disease, School of Public Health, Health Science Center, Xi'an Jiaotong University, Xi’an, Shaanxi, China; 3 Department of Occupational and Environmental Health, School of Public Health, Health Science Center, Xi'an Jiaotong University, No. 76, Yanta West Road, Xi’an, Shaanxi, 710061, China, 86 02982655106; 4 Department of General Dentistry, Jinan Stomatological Hospital, Jinan, China; 5 Department of Health Science Center Global Health Institute, Health Science Center, Xi'an Jiaotong University, Xi’an, Shaanxi, China

**Keywords:** aged, internet medicine, mediation effect, social ecological model, technology acceptance model

## Abstract

**Background:**

The rapid growth of internet health care (IH) offers older adults convenient medical services like remote consultations and health monitoring. However, its adoption among this group remains low, highlighting a significant digital divide. Understanding the behavioral patterns and determinants of IH use in the older population is crucial for optimizing digital health design and improving service accessibility.

**Objective:**

This study aimed to analyze the multidimensional influencing factors of Chinese older adults’ use of IH services based on the integrated framework of the technology acceptance model and social ecological model, and explore their behavioral patterns and key driving factors.

**Methods:**

A cross-sectional study design was adopted to conduct a multistage stratified cluster random sampling survey in 3 cities in Shandong Province from May 2024 to July 2024, with a total of 1828 older adults aged 60 to 75 years included. The study uses latent category analysis to classify the use of IH service behaviors and employs multiple logistic regression, decision tree models, and structural equation modeling to analyze influencing factors and mediating pathways.

**Results:**

Five distinct user groups were identified: nonusers (n=911), registration-dominant users (n=286), low-activity users (n=320), moderate comprehensive users (n=288), and full-service users (n=23). Multinomial logistic regression with nonusers as the reference group identified key determinants: individuals with below primary education had 96% lower odds of membership (odds ratios [OR] 0.039, 95% CI 0.012‐0.084) compared to the reference group with junior college education or above in moderate comprehensive users, while male participants had higher odds of being full-service (OR 1.980, 95% CI 1.126‐3.514) or moderate comprehensive (OR 1.310, 95% CI 1.012‐1.705) users. Older age was consistently associated with lower adoption across all classes. Full-service users exhibited exceptionally high social support (OR 4.502, 95% CI 3.601‐5.627), while moderate comprehensive users showed the highest technology acceptance (OR 2.803, 95% CI 2.355‐3.342). The decision tree model (area under the curve of 0.94) found the optimal path: sufficient social support (≥2), good health status (>5), and high technical acceptance (≥30) yield the highest use probability (92%→96%). Mediation analysis indicated that social support influences usage willingness through both direct and indirect pathways. The direct effect was 0.712 (95% CI 0.552‐0.972; *P*<.001). Among indirect pathways, technology availability and practicality accounted for the largest proportion of mediation (19.7%, 95% CI 16.8%‐22.6%), followed by technology acceptance (13.7%, 95% CI 11.1%‐16.3%) and social influence (8.9%, 95% CI 6.9%‐10.9%).

**Conclusions:**

Optimizing age-friendly design, strengthening social support networks, and improving technological usability are keys to increasing the adoption of IH services among the older population. Future policies should develop targeted intervention strategies for different user groups to narrow the digital health divide.

## Introduction

The rapid development of internet health care (IH) has revolutionized health care delivery, particularly for aging populations [1,2]. IH enhances access to teleconsultations for chronic disease management and enables real-time remote monitoring of vital signs through wearable devices [3-5]. These innovations streamline access to everyday health care tasks, such as teleappointments, online prescription refills, and contactless payment, while enhancing medication adherence through user-friendly digital reminders, reducing the logistical barriers to care for older adults with complex needs [6,7]. However, older adults’ adoption of IH services remains suboptimal, with usage rates and digital literacy gaps perpetuating health inequities [8].

Due to the decline of the older adults’ physiological function, the decline of cognitive flexibility, the lack of digital literacy, and the transformation of social roles, their internet use research has a unique feature of multidimensional interaction and intergenerational cultural conflict [9-12]. These characteristics present particular challenges during the transition to digitization. A study by Yang [11] revealed that perceived usefulness, perceived ease of use, and social influence positively influence older adults’ behavioral intention toward health care services. This finding is consistent with recent extensions of the technology acceptance model (TAM) in digital health contexts, such as the work by Mouloudj et al [12], which validated the core TAM constructs (perceived usefulness and ease of use) in predicting intentions to use digital dental health services, while also highlighting the additional roles of trust and social influence. Meraya’s [13] findings indicated that the primary barriers to telemedicine use were disinclination towards the technology and difficulties in scheduling appointments. Tan’s [14] study underscores the significance of addressing older adults’ subjective well-being and enhancing the accessibility of IH services. While existing studies have significantly advanced our understanding of individual determinants in IH adoption, the complex interplay of technological, psychological, and social factors underscores the need for integrated frameworks to fully capture older adults-specific digital disparities. This multidimensional complexity requires a theoretical framework that simultaneously addresses individual technology perception and macro background influences. The TAM operates on an individual level of adoption through dimensions such as perceived technology usefulness and ease of use, capturing cognitive assessments of technology utility [15].

However, the adoption of digital health technologies among older adults is a process shaped by a complex interplay of individual cognition and multilayered environmental influences. While the TAM effectively explains individual-level cognitive mechanisms through perceived usefulness and ease of use, its limited consideration of broader social contexts necessitates integration with the social ecological model (SEM). The SEM systematically categorizes environmental determinants across interpersonal, community, and policy levels, and this integration creates a conceptually synergistic framework wherein factors at these outer levels actively shape the core TAM constructs. This is particularly salient for older adults who often face unique systemic and interpersonal barriers to technology adoption. Specifically, at the interpersonal level, social support and family recommendations not only enhance perceived usefulness through endorsement but also directly help overcome practical usage barriers, thereby reinforcing perceived ease of use [16,17]. At the community level, resources such as age-friendly training programs and accessible digital infrastructure systematically reduce technical and accessibility barriers, transforming potential obstacles into facilitators of technology acceptance [18]. This combined TAM-SEM framework thus unifies macro-level environmental embeddedness with microlevel cognitive processes, providing a comprehensive theoretical lens to understand and optimize IH accessibility for the older population.

This study synthesizes the TAM and the SEM into a unified theoretical framework, establishing a multidimensional analytical structure to systematically investigate intelligent health technology adoption among older adults. The proposed model systematically integrates individual-level determinants such as electronic health literacy and self-efficacy, interpersonal dynamics exemplified by social support networks, and environmental dimensions encompassing perceived security risks and service accessibility. This tripartite integration enables a stratified exploration of adoption mechanisms, effectively delineating how cognitive-behavioral predispositions, relational influences, and systemic enablers collectively shape technology adoption trajectories within geriatric populations.

The primary aim of this study was to characterize patterns of IH adoption among older adults and to determine the extent to which these patterns can be explained by an integrated framework combining constructs from the SEM and the TAM. We hypothesized that older adults would exhibit distinct, empirically identifiable adoption trajectories, ranging from nonuse to comprehensive engagement. The secondary aims were to examine how a combined SEM–TAM framework predicts IH adoption behavior among older adults. We hypothesized that integrating SEM-based contextual determinants (such as social support and health status) with TAM-based cognitive determinants (such as technology acceptance and perceived usability) would provide a coherent basis for understanding and predicting IH adoption patterns within this population.

## Methods

### Study Population

This study employed a multistage stratified cluster random sampling design to investigate IH service use among older adults aged 60 to 75 in Shandong Province, China. To ensure regional representation, 3 prefecture-level cities representing medium gross domestic product rankings were selected. From each city, we randomly selected 2 counties (representing rural areas) and 2 districts (representing urban areas), achieving a 20% sampling rate at this stage. Within each selected county or district, 2 villages or communities were further chosen through simple random sampling.

Potential participants were identified through local household registry systems. The inclusion criteria required participants to be (1) aged 60 to 75 years, (2) Chinese nationals, and (3) capable of reading and writing. Exclusion criteria included (1) transient residents and (2) individuals with cognitive impairments. Specifically, we excluded those who reported a physician diagnosis of dementia. Additionally, to ensure data quality, we implemented an objective cognitive assessment protocol adapted from standard cognitive screening principles. Exclusion was applied when participants demonstrated (a) failure to comprehend the study purpose and informed consent after 3 structured explanations; (b) inability to answer basic orientation questions (current age, season, or city); or (c) evident disorientation to time or place as evaluated by trained interviewers [19,20].

Between May and July 2024, trained investigators from the Shandong Provincial Center for Disease Control and Prevention conducted face-to-face household interviews. The investigation team consisted of public health professionals with backgrounds in epidemiology, preventive medicine, and health statistics, all of whom had received standardized training on the survey protocol, interview techniques, and ethical considerations specific to this study. A validated questionnaire integrating the TAM and SEM constructs was used. The questionnaire assessed IH service use patterns, perceived ease of use, social support, and environmental barriers. From 1950 eligible individuals approached, 1828 completed valid questionnaires, yielding a response rate of 93.7% (n=1828). The final sample consisted of 48.6% (890/1828) female participants and 52.7% (964/1828) rural residents.

### Ethical Considerations

This study was conducted in accordance with the Declaration of Helsinki and was reviewed and approved by the Medical Ethics Review Committee of the Shandong Provincial Center for Disease Control and Prevention (approval SDJK (K) 2024-046-01). Written or verbal informed consent was obtained from all participants prior to their involvement in the study. As a token of appreciation for their time, participants were provided with a small financial compensation of 10 Chinese Yuan (US $ 1.4). To protect participants’ privacy and ensure confidentiality, all collected data were anonymized and stored securely, with access restricted to the research team. The reporting of this cross-sectional research followed the STROBE (Strengthening the Reporting of Observational Studies in Epidemiology) guideline [21] (Table S1 and Figure S1 in Multimedia Appendix 1).

### Outcome

The primary outcome of IH services use was assessed through a multidimensional approach capturing different aspects of service adoption. Initial screening determined any prior IH use through a binary (yes or no) response to the question “have you used IH services before?” Further use was captured through 2 complementary measures: service breadth, represented by the count of different IH service types used from a comprehensive list of 10 specific services; and user typology, derived via latent class analysis of the specific service use patterns.

The secondary outcome, willingness to use IH in the future, was measured as an indicator of behavioral intention, an established precursor to technology adoption in acceptance theories. This construct was assessed using a 5-point Likert scale in response to the question, “Would you be willing to consider using IH services at your next medical visit?” This approach enables the examination of psychological precursors to adoption alongside actual use behavior within the observational study context.

### Exposure Factors and Covariates

In this study, basic demographic information was collected from participants through a questionnaire. The participants’ educational attainment was categorized as follows: below primary school, primary school, secondary school, high school or technical secondary school, junior college, or above. The marital status of the participants was also documented, with categories including married and unmarried. The participants’ place of residence was categorized as either urban or rural. Key constructs, including usability, self-efficacy, eHealth literacy, perceived risks, social influence, and technology acceptance, were measured using validated scales adapted to the IH context. Usability was assessed using a 6-item adapted System Usability Scale, while self-efficacy was assessed using a 3-item adapted General Self-Efficacy Scale. The eHealth literacy construct was measured using the eHealth scale, and perceived risks were evaluated through a 4-item scale based on the Health Belief Model. Technology acceptance was captured using an 8-item scale derived from the TAM. Social influence was assessed as the degree to which people around me believe I should use IH services. It was measured using the Social Impact Scale [22]. All scales used 5-point Likert-type response formats. Complete measurement details, including all scale items, reliability metrics, and validity evidence, are provided in Table S2 in Multimedia Appendix 1.

### Conceptual Framework

Our research used a conceptual framework that systematically integrated the TAM and the SEM, with variable selection directly informed by the theoretical constructs of both models. This integrated framework specified that technology adoption (the core outcome in TAM) was influenced not only by individual-level factors but also by multilevel social ecological determinants, thus providing the theoretical rationale for our variable selection. The chosen variables mapped directly onto specific theoretical constructs: usability and technology acceptance represented core TAM dimensions capturing perceived ease of use and behavioral intention; self-efficacy and eHealth literacy constituted individual-level factors in the SEM that influenced technology adoption capabilities; perceived risks extended the TAM framework by incorporating threat appraisal mechanisms; social impact and social support operationalized the interpersonal level of the SEM, reflecting how social norms and relational resources affect adoption decisions; and health condition represented an individual-level factor in the SEM that shaped technology adoption capacity and motivation. Notably, “peer influence,” a specific manifestation of social impact, theoretically operated at the interpersonal level of the SEM while simultaneously shaping the core TAM constructs of perceived usefulness and ease of use through social validation and practical assistance. This theoretical mapping explained why social factors demonstrated both direct effects on adoption and indirect effects through technology acceptance pathways in our analyses. The SEM’s levels were intertwined and interactive, with interaction between the older adults and new medical technology occurring in societal (interpersonal) collaboration, all contextualized within health care system environments (Figure S2 in Multimedia Appendix 1) [15,23,24]. This theoretical grounding ensures our variable selection comprehensively captured the multidimensional nature of technology adoption across individual, interpersonal, and community levels.

### Statistical Analysis

**Figure 1.**
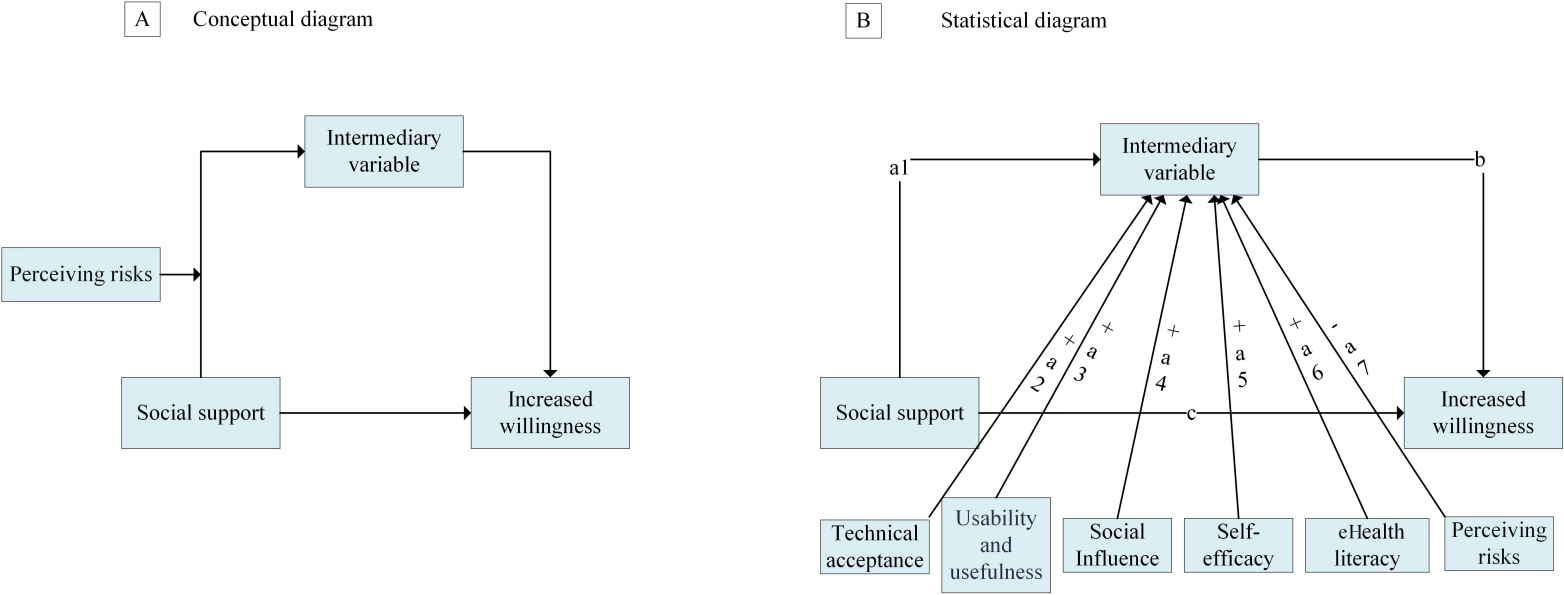
Hypothesized pathways between high technical acceptance, mediating effects, and the health literacy group. (A) Conceptual diagram and (B) statistical diagram.

Latent class analysis (LCA) implemented in *R* classified participants into 5 IH service utilization profiles, optimized via Bayesian information criterion and Akaike information criterion [25]. The resulting classes were subsequently labeled according to their dominant use patterns for intuitive interpretation: class 1: “low-activity testers” (limited exploration of basic functions), class 2: “full-service users” (high probability of using all service types), class 3: “registration-dominant users” (highly focused on appointment services), class 4: “nonusers” (negligible use across all services), and class 5: “moderate comprehensive users” (balanced use of multiple core services). The 5-class solution was retained despite the small sample size (n=23) in class 2 (“full-service users”) due to its strong theoretical relevance as a unique profile of early adopters, characterized by high education, income, and technological proficiency. This group is critical for understanding the full spectrum of digital health adoption. These descriptive labels were used in all subsequent tables and figures to replace numerical codes, enhancing the readability and direct interpretability of the findings. The characteristic use patterns of each class were visualized in the radar chart (Figure S3 in Multimedia Appendix 1). Multinomial logistic regression with elastic net regularization (λ=0.1, *α*=.5) identified predictors of class membership, contrasting nonusers (class 4) against other classes (classes 1‐3, 5) while adjusting for age, health status, and behavioral covariates. A classification and regression tree algorithm with 10-fold cross-validation, trained on an 80:20 stratified split, predicted IH adoption using feature-selected predictors. Moderated mediation pathways were tested via structural equation modeling with robust maximum likelihood estimation, evaluating indirect effects and moderation by perceived risk. Analyses were supported by nonparametric bootstrap resampling (5000 iterations) for bias-corrected confidence intervals. Analyses used *R* packages tidy (LCA), caret classification and regression tree, and lavaan (structural equation modeling), with model diagnostics confirming adequacy (comparative fit index as 0.921, root mean square error of approximation as 0.043, and standardized root mean square residual as 0.049) [26] (Figure 1). All statistical analyses were conducted using R software (version 4.3.1).

## Results

### Patterns and Characteristics of IH Service Adoption Among Older Adults

Among the 1828 respondents, 42.2% (n=772) reported using online appointment registration, 29.7% (n=543) used online payment, and 22.4% (n=410) checked test results online, whereas fewer engaged in online consultations (n=271, 14.8%), test scheduling (n=343, 18.8%), or advanced services such as psychological counseling (n=33, 1.8%), medication delivery (n=39, 2.1%), vaccination appointments (n=109, 6.0%), and in-home care (n=28, 1.5%).

### Characteristics of Crowd Classification

We used LCA models, stratifying participants into 5 IH use classes (Tables S3 and S4 in Multimedia Appendix 1). Radar/Sankey plots visualized use patterns across clusters (Figures S3 and S4 in Multimedia Appendix 1). Low-activity users (class 1, n=320) demonstrated moderate education (n=161, 50.31%) and high technical acceptance (mean 33.72, SD 3.95), primarily engaging in basic functions like online payment. Compared to nonusers (class 4, n=911), they were younger (age: 65.9 y vs 69.0 y). Full-service users (class 2, n=23) featured high education (n=10, 43.48%), urban residence, male predominance (n=15, 65.22%), superior technical adaptability (mean 38.34, SD 4.77), service availability (mean 24.57, SD 3.25), and the highest median income. Registration-dominant users (class 3, n=286) focused on online appointments (>80%), showed moderate technical acceptance (mean 31.22, SD 3.64), lowest perceived risk (mean 10.98, SD 3.32), and male predominance (n=172, 60.14%). Moderate comprehensive users (class 5, N=288) displayed balanced multiservice engagement, with peak technology acceptance (mean 36.17, SD 5.89) and health literacy (mean 11.83, SD 1.67). Key intergroup distinctions involved technology acceptance, eHealth literacy, and urban-rural distribution. Nonusers (class 4, n=911) were predominantly rural (n=566, 62.13%), older (mean 69.0 y), and less educated (n=230, 25.25% ≤primary), and had elevated risk behaviors (n=107, 11.75% smoking every day), indicating a need for targeted interventions (Table 1).

**Table 1.** Baseline characteristics of the study population.

	Total (N=1828)	Class 1 (n=320)^a^	Class 2 (n=23)^a^	Class 3 (n=286)^a^	Class 4 (n=911)^a^	Class 5 (n=288)^a^	*P* value^b^	Relative rank across classes^c^
Sex, n (%)							<.001	
Male	938 (51.31)	158 (49.38)	15 (65.22)	172 (60.14)	426 (46.76)	167 (57.99)		4<1<5<3<2
Female	890 (48.69)	162 (50.63)	8 (34.78)	114 (39.86)	485 (53.24)	121 (42.01)		2<3<5<1<4
Age (years), average (range)	67.9 (60.0‐75.0)	65.9 (60.0‐75.0)	67.2 (60.0‐73.0)	68.2 (60.0‐75.0)	69.0 (60.0‐65.0)	66.6 (60.0‐74.0)	<.001	1<5<2<3<4
Education, n (%)							<.001	
Below primary school	303 (16.58)	22 (6.88)	2 (8.70)	38 (13.29)	230 (25.25)	11 (3.82)		5<1<2<3<4
Primary school	643 (35.18)	63 (19.69)	1 (4.35)	107 (37.41)	355 (38.97)	108 (37.5)		2<1<3<5<4
Secondary school	565 (30.91)	161 (50.31)	5 (21.74)	89 (31.12)	225 (24.7)	85 (29.51)		2<4<5<3<1
High school or technical secondary school	250 (13.68)	57 (17.81)	5 (21.74)	44 (15.38)	85 (9.33)	59 (20.49)		4<3<1<5<2
Junior college or above	67 (3.67)	17 (5.31)	10 (43.48)	8 (2.8)	16 (1.76)	25 (8.68)		4<3<1<5<2
Residency, n (%)							<.001	
Urban	864 (47.26)	165 (51.56)	23 (100)	120 (41.96)	345 (37.87)	211 (73.26)		4<3<1<5<2
Rural	964 (52.74)	155 (48.44)	0 (0)	166 (59.04)	566 (62.13)	77 (26.74)		2<5<1<3<4
Marriage, n (%)							<.001	
No	212 (11.60)	25 (7.81)	2 (8.70)	42 (14.69)	117 (12.84)	26 (9.03)		1<2<5<4<3
Yes	1616 (88.40)	295 (92.19)	21 (91.30)	244 (85.31)	794 (87.16)	262 (90.97)		3<4<5<2<1
Incomes^d^	4.34 (0.67‐26.82)	5.89 (1.42‐22.15)	8.73 (4.24‐24.36)	4.96 (2.17‐15.83)	2.87 (0.67‐9.29)	6.92 (2.94‐18.65)	.16	—^e^
Smoke, n (%)							<.001	
Everyday	196 (10.72)	48 (15.00)	2 (8.70)	18 (6.30)	107 (11.75)	21 (7.29)		3<5<2<4<1
Not everyday	58(3.17)	18 (5.63)	1 (4.35)	22 (7.69)	13 (1.42)	4 (1.39)		5<4<2<1<3
Never	1574 (86.11)	254 (79.37)	20 (86.95)	246 (86.01)	791 (86.83)	263 (91.32)		1<3<4<2<5
Drink, n (%)							<.001	
No	1333 (72.92)	222 (69.38)	20 (86.95)	207 (72.38)	662 (72.67)	222 (77.08)		1<3<4<5<2
Quit drinking	218 (11.93)	38 (11.86)	1 (4.35)	51 (17.82)	108 (11.86)	20 (6.95)		2<5<1<4<3
Yes	277 (15.15)	60 (18.76)	2 (8.70)	28 (9.80)	141 (15.47)	46 (15.97)		2<3<4<5<1
Usability and usefulness, mean (SD)	20.30 (2.47)	22.58 (1.83)	24.57 (3.25)	21.55 (1.98)	17.25 (2.61)	25.83 (2.34)	<.001	4<3<1<2<5
Self-efficacy, mean (SD)	9.53 (1.72)	11.14 (1.56)	11.61 (3.14)	9.67 (1.37)	7.92 (1.89)	12.51 (1.78)	<.001	4<3<1<2<5
Perceiving risks, mean (SD)	12.55 (3.01)	12.54 (2.98)	13.09 (4.56)	10.98 (3.32)	13.41 (3.17)	13.14 (2.89)	<.001	3<2<5<1<4
Electronic health literacy, mean (SD)	9.71 (1.65)	11.20 (1.79)	10.78 (3.82)	10.53 (1.46)	8.23 (2.15)	11.83 (1.67)	<.001	4<3<2<1<5
Social impact, mean (SD)	9.71 (1.88)	11.13 (1.92)	11.48 (3.63)	10.16 (1.54)	8.19 (2.27)	12.34 (1.81)	<.001	4<3<1<2<5
Social support, mean (SD)	2.94 (0.87)	2.35 (0.65)	6.07 (2.43)	2.91 (0.92)	2.00 (0.58)	2.03 (0.69)	<.001	4<5<1<3<2
Health status, mean (SD)	8.70 (1.34)	8.88 (1.17)	9.91 (2.75)	8.65 (1.28)	8.41 (1.43)	9.39 (1.56)	<.001	4<3<1<5<2
eHealth literacy, mean (SD)	9.92 (3.51)	10.78 (2.39)	11.83 (2.28)	10.53 (4.30)	8.23 (3.11)	11.20 (2.86)	<.001	4<3<1<2<5
Technology acceptance, mean (SD)	29.83 (4.12)	33.72 (3.95)	36.17 (5.89)	31.22 (3.64)	25.17 (3.38)	38.34 (4.77)	<.001	4<3<1<2<5

a A total of 5 user classes were identified: class 1 (low-activity tasters, n=320; minimal engagement with select internet health care services), class 2 (full-service users, n=23; high-frequency multifunctional platform use), class 3 (registration-dominant users, n=286; predominant appointment bookings), class 4 (nonusers, n=911; no internet health care adoption), and class 5 (moderate comprehensive users, n=288; intermediate engagement with integrated services, at lower intensity than class 2).

b *P* values were derived in accordance with established methodology, encompassing the chi-square test for categorical variables (eg, sex, education) and the Kruskal-Wallis test for continuous variables (eg, age, income). The significance level was set at .05.

c Classes ranked from lowest to highest value based on descriptive statistics; only shown for variables with *P*<.05.

d The data regarding income are expressed in RMB and the unit is 10,000 yuan (US $1428.6 ), the value is the median (range), rounded to 2 decimal places. Data processed by Winsorize (truncated by 1% extreme values at the beginning and end).

e Not available.

As shown in Table 2, the factors associated with membership in the distinct IH user typologies were derived from a multinomial logistic regression analysis with class 4 (nonusers) as the reference group. The analysis revealed a clear social gradient in IH adoption. This was most evident in class 5 (moderate comprehensive users), where individuals with below primary education had 96% lower odds of membership (OR 0.039, 95% CI 0.012‐0.084) compared to the reference group with junior college education or above. Male gender was a significant predictor of membership in the more engaged classes, specifically class 2 (full-service users; OR 1.980, 95% CI 1.126‐3.514) and class 5 (OR 1.310, 95% CI 1.012‐1.705). Conversely, older age was consistently associated with significantly diminished odds of being in any user class relative to nonusers.

The psychographic and social determinants further delineated the user classes. Class 1 (low-activity tasters) was reported as having higher social impact (OR 1.221, 95% CI 1.125‐1.325) and marginally positive technology acceptance (OR 1.951, 95% CI 1.630‐2.332); their risk perception was not significantly different from nonusers, though they demonstrated moderate eHealth literacy (OR 1.905, 95% CI 1.597‐2.279). Class 2 (full-service users) was distinguished by higher levels of social support (OR 4.502, 95% CI 3.601‐5.627) and technology acceptance (OR 2.302, 95% CI 1.937‐2.759), alongside elevated eHealth literacy (OR 1.729, 95% CI 1.443‐2.060). Class 3 (registration-dominant users) also exhibited significant advantages in social support (OR 1.658, 95% CI 1.381‐1.979) and technology acceptance (OR 1.652, 95% CI 1.314‐1.908) compared to nonusers, complemented by higher eHealth literacy (OR 1.651, 95% CI 1.383‐1.974). Class 5 (moderate comprehensive users) demonstrated the highest odds ratios (ORs) in eHealth literacy (OR 2.202, 95% CI 1.849‐2.633), social impact (OR 1.320, 95% CI 1.194‐1.459), and technology acceptance (OR 2.803, 95% CI 2.355‐3.342).

**Table 2.** Correlates of user typology membership from multivariable logistic regression.^a^

Covariates	Class 1: low-activity tasters, OR^b^ (95% CI)	Class 2: full-service users, OR (95% CI)	Class 3: registration-dominant users, OR (95% CI)	Class 5: moderate comprehensive users, OR (95% CI)
Sex				
Female (reference)	1.0	1.0	1.0	1.0
Male	0.696 (0.474‐1.023)	1.980^c^ (1.126‐3.514)	1.463^c^ (1.110‐1.932)	1.310^c^ (1.012‐1.705)
Age	0.879^c^ (0.846‐0.919)	0.892^c^ (0.790‐0.982)	0.937^c^ (0.903‐0.973)	0.888^c^ (0.846‐0.931)
Residency				
Urban	1.404 (0.963‐2.045)	—^d^	1.182 (0.897‐1.574)	2.663^c^ (1.724‐4.113)
Rural (reference)	1.0	1.0	1.0	1.0
Education				
Below the primary school	0.111^c^ (0.047‐0.191)	0.073^c^ (0.022‐0.142)	0.282^c^ (0.121‐0.667)	0.039^c^ (0.012‐0.084)
Primary school	0.271^c^ (0.151‐0.497)	0.018^c^ (0.002‐0.059)	0.804 (0.367‐1.778)	0.331^c^ (0.202‐0.552)
Secondary school	1.109 (0.611‐3.728)	0.152^c^ (0.095‐0.231)	0.668 (0.297‐1.499)	0.255^c^ (0.157‐0.424)
High school or technical secondary school	0.662 (0.364‐1.211)	0.152^c^ (0.095‐0.231)	0.348^c^ (0.159‐0.786)	0.180^c^ (0.108‐0.329)
Junior college or above (reference)	1.0	1.0	1.0	1.0
Marriage				
No	0.584^c^ (0.372‐0.899)	0.453^c^ (0.211‐0.959)	1.508^c^ (1.108‐2.050)	0.651^c^ (0.430‐0.997)
Yes (reference)	1.0	1.0	1.0	1.0
Smoke				
Everyday	1.402 (0.973‐2.020)	0.745 (0.167‐3.416)	0.457^c^ (0.242‐0.861)	0.642 (0.393‐1.057)
Not everyday	3.803 (2.051‐7.061)	0.393 (0.055‐3.113)	1.866^c^ (1.169‐8.042)	0.120^c^ (0.004‐0.346)
Never (reference)	1.0	1.0	1.0	1.0
Drink				
No	0.529^c^ (0.315‐0.889)	2.621 (0.566‐12.300)	1.338 (0.772‐2.320)	1.275 (0.853‐1.894)
Quit drinking	0.687 (0.351‐1.354)	0.314 (0.114‐7.430)	1.809 (0.934‐3.503)	0.654 (0.293‐1.459)
Yes (reference)	1.0	1.0	1.0	1.0
Usability and usefulness	1.041 (0.994‐1.090)	1.553^c^ (1.107‐2.184)	1.084^c^ (1.038‐1.132)	1.136^c^ (1.077‐1.199)
Self-efficacy	1.103 (0.853‐1.388)	1.811^c^ (1.253‐2.601)	1.012 (0.934‐1.097)	1.971^c^ (1.372‐2.879)
Perceiving risks	0.975 (0.931‐1.022)	0.827^c^ (0.619‐0.981)	0.806^c^ (0.730‐0.904)	0.955^c^ (0.913‐0.999)
Electronic health literacy	1.905^c^ (1.597‐2.279)	1.729^c^ (1.443‐2.060)	1.651^c^ (1.383‐1.974)	2.202^c^ (1.849‐2.633)
Social impact	1.221^c^ (1.125‐1.325)	1.248^c^ (1.071‐1.603)	1.164^c^ (1.078‐1.258)	1.320^c^ (1.194‐1.459)
Social support	1.257^c^ (1.055‐1.496)	4.502^c^ (3.601‐5.627)	1.658^c^ (1.381‐1.979)	1.027 (0.857‐1.229)
Health status	1.201^c^ (1.014‐1.430)	1.853^c^ (1.555‐2.214)	1.108 (0.927‐1.319)	1.451^c^ (1.224‐1.727)
Technology acceptance	1.951^c^ (1.630‐2.332)	2.302^c^ (1.937‐2.759)	1.652^c^ (1.314‐1.908)	2.803^c^ (2.355‐3.342)

a With class 4 (nonusers) as a reference OR value (ratio), OR represents the probability ratio of a certain type of user possessing a certain feature relative to nonusers. OR>1: this type of user is more likely to possess the feature, OR<1: this type of user is less likely to possess the feature. The analysis used multinomial logistic regression with Bonferroni correction for multiple comparisons. Adjusted covariates included sex, age, education level, residency, marital status, income, smoking status, and drinking status. Five user classes were identified: class 1 (low-activity tasters, n=320; minimal engagement with select internet health care services), class 2 (full-service users, n=23; high-frequency multifunctional platform use), class 3 (registration-dominant users, n=286; predominant appointment bookings), class 4 (nonusers, n=911; no internet health care adoption), and class 5 (moderate comprehensive users, n=288; intermediate engagement with integrated services, at lower intensity than class 2).

b OR: odds ratio.

c Statistically significant at the .05 level.

d OR and CI could not be calculated because the model did not converge, likely due to a lack of variation in residency (urban/rural) within class 2.

### Predictive Factors

**Figure 2.**
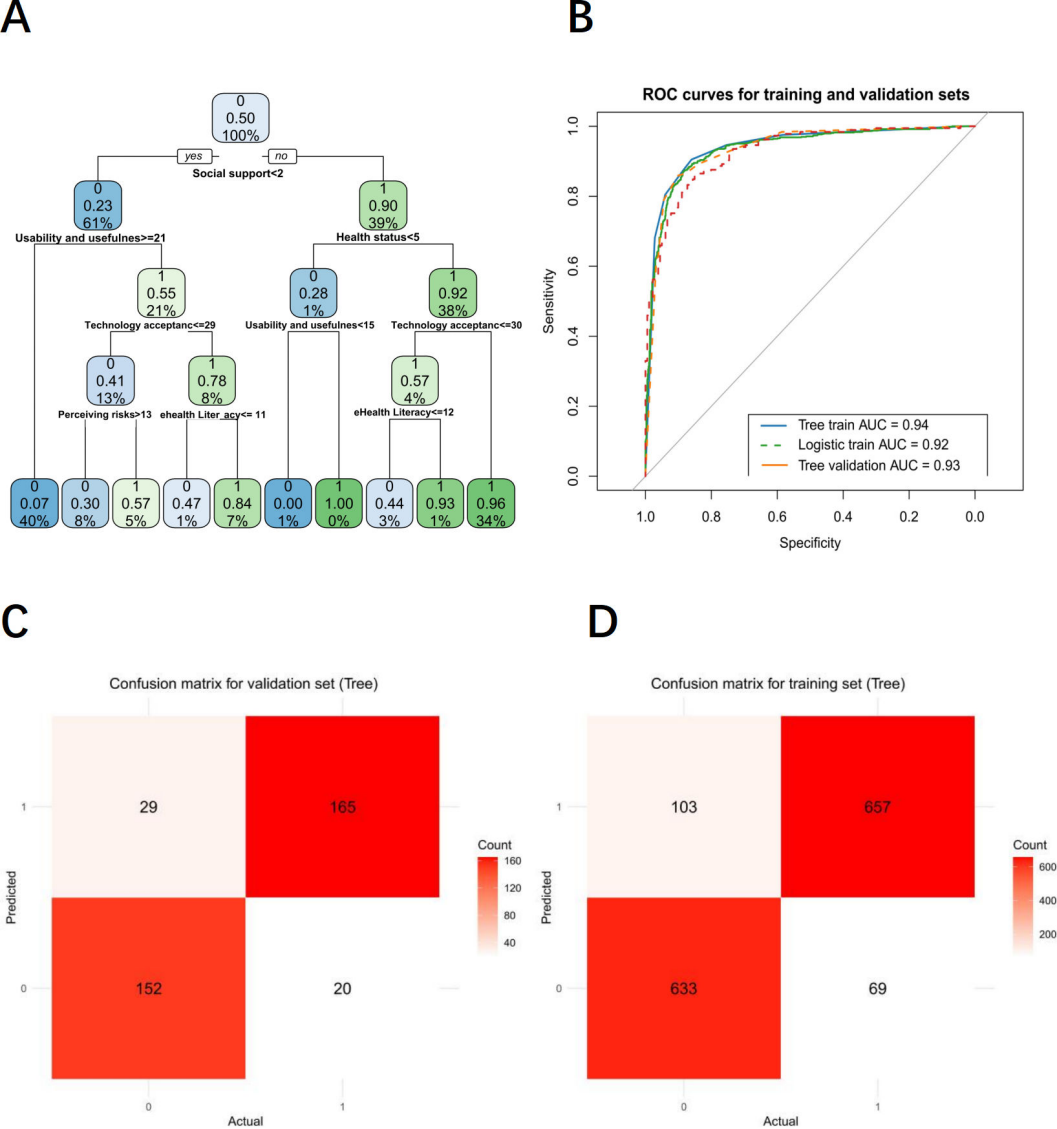
A decision tree model for predicting internet health care service use among older adults. AUC: area under the curve; ROC: receiver operating characteristic.

The model showed high accuracy in classifying future internet use intention, with area under the curve values of 0.94 (training set) and 0.93 (test set) (Figure 2B). Figure 2C and 2D showed the decision tree model’s confusion matrices for the validation and training sets, respectively. Table S5 in Multimedia Appendix 1 shows high sensitivity in both test and training sets, with higher specificity, accuracy, precision, F1 score, lower false positive rate, and good overall test-set performance. The decision tree model revealed the recursive dichotomy of the multivariate feature space affecting IH service use probability. The variable importance order is: social support, health status, usability and usefulness, technology acceptance, perceiving risks, and eHealth literacy. In the left branch, future use probability dropped sharply to 23%. Further division showed that a technology usability and practicality score ≥21 (21% subsample) could partially offset insufficient social support, increasing probability to 55%; but with technology acceptance ≤29 (13% subsample), probability drops to 41%. When perceived health risk >13 (40% subsample), usage probability dropped to 7%, forming the lowest usage group. In the right branch, usage probability jumped to 90%, with health status >5 (38% subsample), further pushing it to 92%, forming a core high-usage group. For those with technology acceptance ≥30 and eHealth literacy ≥12 (34% subsample), use probability reached 96%, reflecting technology and literacy’s multiplier effect. The optimal path: sufficient social support (≥2), good health status (>5), and high technical acceptance (≥30) yielded the highest use probability (92% →96%). Insufficient social support (<2) with high-risk perception (>13) led to extremely low use probability (7%), an 89% point difference from the optimal path (Figure 2).

The direct effect showed that for every 1 unit increase in social support, the willingness to use increased by 0.712 (95% CI 0.552‐0.872; *P*<.001). Among the indirect effects, the mediating contribution of technology usability and practicality is the largest, accounting for 19.7% (95% CI 16.8%‐22.6%) of the total effect, followed by technology acceptance (13.7%, 95% CI 11.1%‐16.3%) and social influence (8.9%, 95% CI: 6.9%‐10.9%). Although self-efficacy (3.2%) and eHealth literacy (2.7%, 95% CI 0.9%‐4.5%) were significant, their contributions are relatively small, while perceived risk was weakly negatively mediated (−0.6%, *β*=−.007) (Table 3).

**Table 3.** Mediation analysis of the effect of social support on willingness for future internet health care use^a^.

Pathway	β (95% CI)	*P* value	Mediation proportion^b^, % (95% CI)
Total effect (social support → willingness to use)	1.198 (1.036 to 1.360)	<.001	100
Direct effect (social support → willingness to use)	0.712^c^ (0.552 to 0.872)	<.001	59.4 (54.1 to 64.7)
Total indirect effect	0.486^c^ (0.420 to 0.552)	<.001	40.6 (35.3 to 45.9)
Mediated pathways (social support → mediator → willingness)			
Usability and usefulness	0.236^c^ (0.205 to 0.267)	<.001	19.7 (16.8 to 22.6)
Technology acceptance	0.164^c^ (0.134 to 0.194)	<.001	13.7 (11.1 to 16.3)
Social influence	0.107^c^ (0.085 to 0.129)	<.001	8.9 (6.9 to 10.9)
Self-efficacy	0.038^c^ (0.021 to 0.055)	<.001	3.2 (1.7 to 4.7)
eHealth literacy	0.032^c^ (0.011 to 0.053)	.004	2.7 (0.9 to 4.5)
Perceived risk	−0.007^c^ (−0.010 to −0.004)	<.001	−0.6 (−0.9 to‐0.3)
Health status	0.009 (−0.002 to 0.020)	.12	—^d^

a Model adjusted variables: all analyses adjusted for sex, age, education level, place of residence (urban-rural), marital status, income level, and chronic disease status. Mediation effect calculation: the bias-corrected bootstrap method (5000 repeated samples) is used to estimate the indirect effect. Path explanation: Direct effect: independent impact of social support on usage intention (without mediating variables). Indirect effect: The chain effect of social support on usage intention through mediating variables.

b The formula for calculating the mediation ratio is: indirect effect/(direct effect + indirect effect) × 100%.

c *P*<.001; a 95% CI that is non-zero is considered significant.

d Not applicable.

## Discussion

### Principal Findings

This study divides the use of IH services by older adults into 5 groups through cluster analysis and constructs an integrated analysis framework based on ecological and technological models, providing a multidimensional perspective for understanding the complex mechanisms of older adults’ use of IH services. Research has found that the overall adoption rate of IH services among the elderly population is low, with nearly half of the older adults never using related services and only a very small number becoming regular users. Multivariate analysis shows that advanced age, psychosocial factors, and ease of use of technology are key predictive factors affecting IH participation. Age growth and daily material use are negatively correlated with IH use, while technology usability awareness and electronic health literacy significantly promote usage behavior. Social support drives usage behavior through both direct effects (increasing willingness to use) and indirect effects (mediating effects of technology availability, technology acceptance, and social impact), with the mediating contribution of technology availability being the most prominent. The study emphasizes the need to restructure the design and policy framework of IH services to address the social and technological needs of the older population, in order to bridge the intergenerational gap in the use of health technology.

### Theoretical Interpretation of User Patterns Through TAM-SEM Integration

The 5 distinct user patterns emerging from our analysis can be effectively interpreted through our integrated theoretical framework, with the SEM elucidating how macro-level social structures regulate microlevel behavioral patterns. Consistent with prior research on technology adoption barriers [11,27], the low-activity testers (class 1), despite demonstrating moderate technical acceptance, exhibited limited engagement, reflecting how high perceived risk (a TAM extension) and insufficient reinforcing factors at the interpersonal level of the SEM can inhibit adoption even when basic ease of use is acknowledged. This pattern aligns with studies highlighting the critical role of privacy concerns in limiting technology engagement among vulnerable populations [28]. Conversely, the full-service users (class 2), though small in number, exemplify the synergistic effect postulated by the SEM. However, the exceptionally low proportion of comprehensive users in our Chinese sample contrasts sharply with reports from high-income countries [29], suggesting contextual moderators, including structural barriers and cultural preferences for traditional care.

The registration-dominant users (class 3) present a compelling case where a specific, high-use function drives adoption despite moderate overall technical acceptance, echoing previous observations of “feature-specific adoption” in health technology literature [30]. This pattern provides empirical support for the “minimum viable product” approach advocated in implementation science, while simultaneously revealing its limitations in fostering broader platform engagement. Meanwhile, the moderate comprehensive users (class 5) demonstrate how optimized perceptions across multiple TAM dimensions facilitate broader adoption, reinforcing established technology acceptance theories while extending them to the geriatric context [31]. The nonusers (class 4) represent a population where deficiencies across multiple SEM levels create compounded barriers, a finding that resonates with digital divide research across socioeconomic strata. This multilevel barrier pattern aligns with Bronfenbrenner’s ecological systems theory while highlighting its specific manifestations in digital health contexts [32].

### TAM-SEM Predictors

The introduction of technical models further reveals the data-driven prediction mechanism. The decision tree model identifies 6 core predictors of older adults’ future willingness to use IH services. While perceived risk demonstrates a significant moderating effect that weakens the impact of technology usability on use frequency, usability and usefulness emerge as equally crucial factors that directly facilitate adoption through enhancing perceived ease of use. This finding extends beyond the risk-focused narrative prevalent in digital health literature, revealing a more balanced interplay between barriers and facilitators [33]. Cross-cultural comparisons further illuminate these relationships. Our findings regarding the strong predictive power of usability align with Möller et al’s [34] European-Japanese study documenting how interface design fundamentally shapes older adults’ digital engagement patterns. However, whereas their research emphasized technological coaching systems, our model reveals how usability interacts with locally specific factors like family support networks in the Chinese context. Similarly, Morishita-Suzuki et al [35] identified depression and leisure activities as crucial determinants of eHealth literacy across European Union and Japanese older adults, while our study extends this understanding by quantifying how eHealth literacy functions within a broader predictive framework alongside usability and social support. The identified predictor hierarchy—social support, health status, usability and usefulness, technology acceptance, eHealth literacy, and perceived risks—represents a significant departure from the conventional TAM that typically prioritizes cognitive perceptions over contextual factors. This corresponds to the “perceived usefulness → usage behavior” pathway in the TAM framework, where perceived usability directly affects technology adoption, while social support indirectly enhances usage intention through a mediating pathway that alleviates anxiety. The prominent role of usability in our model underscores its fundamental importance in gerontechnology design, particularly for populations with limited digital experience. This dual-framework approach reveals that social support not only directly encourages adoption but also indirectly facilitates use through enhancing technology acceptance and reducing anxiety, while usability serves as the foundational element that makes initial engagement possible for older users. This suggests that technology design needs to prioritize addressing pain points such as privacy protection and operational transparency to reduce decision-making barriers for older users [36-38]. This dual-framework approach reveals that social support not only directly encourages adoption but also indirectly facilitates usage through enhancing technology acceptance and reducing anxiety.

### Digital Inequality and Policy Implications

This study also underscores the role of digital inequality and broader social determinants. Limited access to smartphones, poor connectivity in rural communities, and lower education levels all constrained adoption, reflecting entrenched disparities in digital health equity. Such inequalities suggest that IH adoption cannot be understood solely as an individual decision but rather as an outcome shaped by structural opportunities and constraints. Addressing these challenges requires coordinated policies that integrate infrastructure investment, targeted subsidies, and the inclusion of IH capacity indicators in routine health assessments [37,38].

The cross-analysis of the 2 models shows that moderate comprehensive service users (class 5) are more sensitive to the usability of the technical interface, while registered dominant users (class 3) are limited to the singular use of service functions, reflecting the gap between the functional integration of the IH platform and the matching needs of user segmentation. From the perspective of policy practice, the inspiration of the ecological model lies in constructing a 3-dimensional intervention system of “individual capability enhancement, social support strengthening, technological environment adaptation” [39,40]: for nonservice users (class 4), it is necessary to reduce perceived risks through community digital literacy training; for registered dominant users, the flow design of appointment services and other functions should be optimized to promote the transformation from a single service to comprehensive use. The successful cases of full-service users provide empirical evidence for the “technology feedback” family model, which amplifies the positive effects of social support through intergenerational collaboration mechanisms. The application value of technical models lies in accurately identifying high-risk groups, such as daily smokers and the older population, who account for a higher proportion of non–service users. Hierarchical intervention can be carried out through the risk scores output by decision tree models, combined with simulation training and privacy protection technology, gradually building an inclusive digital health ecosystem [41,42].

To implement these insights, we propose a further intervention strategy. At the microlevel, develop TAM-driven “cognitive scaffolding” interfaces, such as AI voice assistants with gerontologically optimized dialogue flow, to reduce navigation complexity. Enforce the IH platform to implement general data protection regulation privacy dashboards using intuitive icons, directly addressing the perceived risks we identified in risk literacy balance. At the macro level: standardize algorithm governance through the “elderly friction” policy—forcing IH platforms to disable autoplay functionality and insert reflective prompts (such as “You browsed for 10 min—take a break!”) to prevent forced use [43]. Incorporate IH capability indicators into the national elderly health assessment, prioritizing infrastructure upgrades in areas with a lower prevalence of level 2 users [44]. Establish “Digital Health Hub” Peer-Learning Models: community centers (eg, libraries and senior clubs) should host regular peer-led training sessions, facilitated by trained “Digital Health Champions” from among tech-savvy seniors [45].

The combined application of ecological and technological models also highlights the originality of this study. While ecological models emphasize external environments and TAM focuses on perceptions of usability, their integration offers a multidimensional explanation of adoption. This dual perspective enhances the explanatory power of our findings and demonstrates that interventions must simultaneously target cognitive, relational, and systemic barriers to promote digital health use among older adults.

### Limitations

There are several limitations that should be acknowledged in this study. First, a cross-sectional design limits causal reasoning. Although the relationship between the identified factors (such as social support and technology acceptance) and the use of IH services has been observed, a longitudinal study is needed to establish a time relationship and determine whether the improvement of social support and technology acceptance will lead to an increase in the IH adoption rate over time. Second, due to regional sampling, its generalizability is limited. This regional focus may limit the generalizability of research results to other socio-economic backgrounds or geographical regions. Third, the screening of participants for cognitive impairment was based on prior research and clinical judgment rather than a standardized instrument like the Mini-Mental State Examination. While this approach was pragmatic for our large-scale study, it may have resulted in some residual confounding. Fourth, our scale was adapted from a standardized instrument based on the actual conditions of older adults in China, which may affect its external generalizability. These limitations highlight important directions for future research, including longitudinal design, broader geographic sampling, inclusion of objective use data, and a more comprehensive assessment of background factors influencing the adoption of IH in the older population.

### Conclusions

This study uses an integrated TAM and SEM framework to examine IH service use among older adults in China. The identification of 5 distinct user profiles demonstrates substantial diversity in adoption patterns, ranging from complete nonuse to comprehensive adoption. Findings reveal that technology adoption is shaped by a multifaceted interplay of cognitive perceptions, relational resources, and systemic enablers. Willingness to use serves as a crucial psychological precursor to actual adoption, influenced by both technological factors and social-environmental contexts. Usability and perceived risk represent significant technological determinants, while social support facilitates adoption through multiple pathways. The integrated TAM-SEM framework provides theoretical value by demonstrating how factors across ecological levels collectively shape adoption behaviors. For practical application, technology design should prioritize aging-friendly interfaces and privacy protections, while community programs should strengthen social support through tailored digital literacy initiatives. Policy efforts must address structural barriers in underserved areas. Future research should explore the longitudinal evolution of user profiles across diverse contexts. By developing targeted strategies for different user segments, we can foster an inclusive digital health ecosystem that benefits all older adults regardless of technological proficiency or social resources.

## Supplementary material

10.2196/78037 Multimedia Appendix 1 Study design, measures, and analytic visuals related to internet use among older adults.

10.2196/78037 Checklist 1 STROBE checklist.

## Abbreviations

IH: internet health care
LCA: latent class analysis
OR: odds ratio
SEM: social ecological model
STROBE: Strengthening the Reporting of Observational Studies in Epidemiology
TAM: technology acceptance model
