# Supplementary material for: Internet Health Care Service Use Behavioral Pattern Among Older Adults and the Role of the Technology Acceptance and Social Ecological Theory Model: Cross-Sectional Survey
Source: J Med Internet Res. 2026 Jan 15;28:e78037. doi: 10.2196/78037 (PMC12806595; doi:10.2196/78037)
Supplement: Multimedia Appendix 1 [file jmir-v28-e78037-s001.docx]

Supplementary material

**Figure S1. Sample Research and Screening Process Diagram**


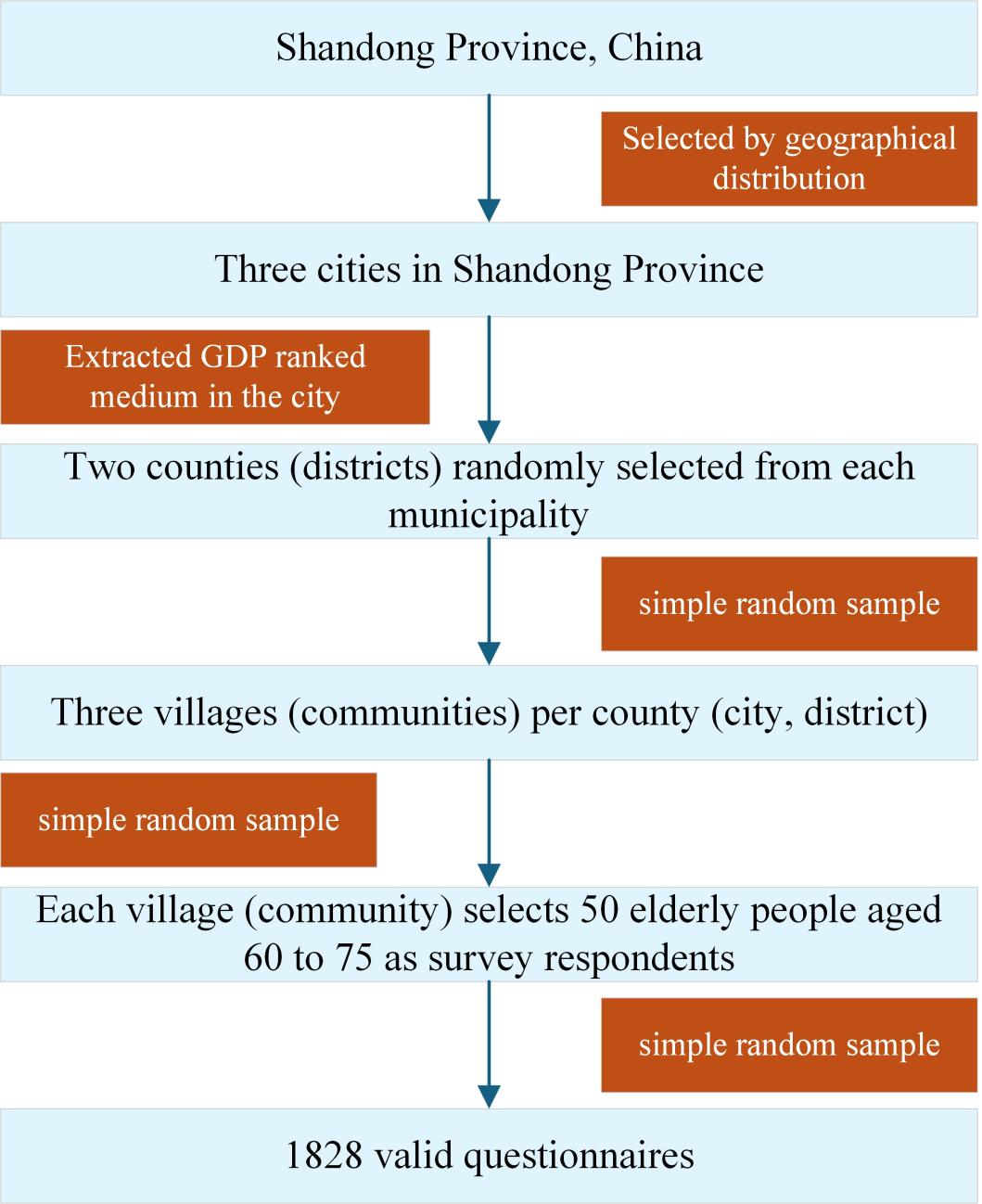


**Table S1. Theme Explain the Impact of Internet Use by older adults**

| Theme and subtheme | Exemplar quotation |
| --- | --- |
| Theme 1: Low-activity service testers | |
| Subthemes | |
| Partial online medical services | We often use online payment for medical expenses. |
|  | We don't know how online counseling and online appointments for in-home care work. |
| Function Attempt Threshold | We were concerned that counseling and in-home care services would compromise our privacy. |
| Theme 2: Comprehensive service users | |
| Subthemes | |
| Efficient and simple | The use of online medical services can be very good to help us review the full of the disease, and the delivery of medication is also faster. |
|  | We can easily see the results of our tests online, and the doctors provide us with online interpretation services. |
| Behavioral motivation | My children taught me how to use online healthcare during the epidemic. |
|  | Online medical service interface launched for senior citizens to facilitate our operation. |
| Theme 3: Registered dominant user | |
| Subthemes | |
| Demand-driven | In our daily use we find it difficult to register offline and easy to register online. |
|  | Immediately after we completed our registration and moved to the offline process, we didn't realize that we could also check lab results online |
| Cognitive limitation | To us internet healthcare is the appointment booking tool that allows us to pick the doctor we want to see without having to go to the hospital. |
| Theme 4: Non-service user | |
| Subthemes | |
| Digital divide | I don't have a cell phone. |
|  | Our village has a poor internet signal. |
| Confidence deficit | Are e-prescribing and online doctor consultations reliable? |
|  | I'm used to “face-to-face” treatment, I can't describe what's wrong from the screen, and I have to go to the hospital in the end. |
| Theme 5: Medium users of integrated services | |
| Subthemes | |
| Value identity | Online registration, report checking and payment preference can reduce offline waiting time. |
|  | We recognize the convenience of basic services, but don't want to pay extra for value-added services. |
| Risk avoidance | The online medical registration is enough, the consultation always makes me feel inaccurate. |

**Table S2：Measurement Scales and References for Key Study Indicators**

| Indicator | Scale Name | Measurement Method | Design Reference |
| --- | --- | --- | --- |
| Usability | System Usability Scale (SUS) (adapted) [1] | 6 items, 5-point Likert scale (1=Strongly disagree, 5=Strongly agree) Total score range: 6–30 (higher scores = higher usability/usefulness) | **Items**: 1. Useful in my daily life 2. Easy to operate and can be used immediately 3. Friendly design, easy to find needed functions in the system (6 items total, covering core SUS dimensions);  **Psychometrics**: -Reliability: Cronbach’s α = 0.89 -Validity: Exploratory factor analysis (EFA) showed a unidimensional structure, factor loadings ranged 0.72–0.84 (e.g., "Easy to operate" = 0.84, "Indispensable medical access" = 0.72), |
| Self-efficacy | General Self-Efficacy Scale (GSES) (adapted) [2] | 3 items, 5-point Likert scale (1=Very inconsistent, 5=Very consistent) Total score range: 3–15 (higher scores = stronger self-efficacy in using internet healthcare) | **Items**(promoting internet healthcare operation): 1. Can complete the task if someone teaches me how 2. Can complete the task if given enough time to operate 3. Can complete specified operations even without prior experience with similar systems (3 items total, adapted for internet healthcare context);  **Psychometrics**: -Reliability: - Cronbach’s α = 0.948 - Corrected Item-Total Correlation (CITC): 0.870–0.921 (all > 0.5, indicating strong item-scale correlation) |
| Perceived risks | Health Belief Model (HBM) Risk Scale | 4 items, 5-point Likert scale (1=Strongly disagree, 5=Strongly agree) Total score: 4–20 (higher = stronger risk perception) | **Items**: 1. Risk of personal privacy/health data leakage 2. Risk of property security during payment 3. Concern about insufficient communication with doctors and poor service quality 4. Worry about fraudulent information and deception  **Psychometrics**(adapted for internet healthcare): - Cronbach’s α = 0.88 (estimated for similar risk scales) - Factor loadings: 0.72–0.89 (items loading onto a single "perceived risk" factor) |
| E-health literacy | eHealth Literacy Scale (eHEALS) (adapted) [3, 4] | 3 items, 5-point Likert scale (1=Strongly disagree, 5=Strongly agree) Total score: 3–15 (higher = higher literacy) | **Items**(examples): 1. I know how to use online health information to help myself 2. I can evaluate the quality of online health resources 3. I am confident in using online information for health decisions   **Psychometrics**: - Cronbach’s α = 0.89 (original scale) - CITC: 0.65–0.81 - Confirmatory factor analysis: one-factor solution (CFI=0.92, RMSEA=0.06) |
| **Social Impact** | Social Impact Scale (custom-adapted) [5] | 5 items, 5-point Likert scale (1=Strongly disagree, 5=Strongly agree) Total score: 5–25 (higher = greater social influence) | **Items**(5 total): 1. People around me think I should learn to use internet healthcare 2. People around me generally use internet healthcare 3. Others consider me capable when I use internet healthcare 4. My social circle encourages me to use online medical services 5. Using internet healthcare affects my social reputation  **Psychometrics**: - Exploratory factor analysis: single-factor structure - Cronbach’s α = 0.82 (adapted version) |
| Health condition | Self-Rated Health Scale (adapted) [6] | Single-item, 11-point scale (0=Worst health, 10=Best health) | **Item**: "Please rate your current health status (0–10, where 10 is excellent)" |
| **Technology Acceptance** | Technology Acceptance Model (TAM) Scale [7] | 8 items, 5-point Likert scale (1=Strongly disagree, 5=Strongly agree) Total score: 8–40 (higher = higher acceptance) | **Items**(examples from TAM): 1. Using internet healthcare services is useful for my health management 2. I find it easy to use internet healthcare platforms 3. I would recommend internet healthcare to others 4. Using internet healthcare saves me time 5. The interface of internet healthcare is user-friendly (8 items total, covering perceived usefulness, ease of use, and behavioral intention)  **Psychometrics**(original TAM): - Cronbach’s α = 0.89 - Factor loadings: 0.75–0.91 (two-factor structure: usefulness & ease of use) |

**References**

1. Byrd TF 4th, Kim JS, Yeh C, Lee J, O'Leary KJ. Technology acceptance and critical mass: Development of a consolidated model to explain the actual use of mobile health care communication tools. J Biomed Inform. 2021;117:103749. doi:10.1016/j.jbi.2021.103749

2. Chen K, Chan AH. Gerontechnology acceptance by elderly Hong Kong Chinese: a senior technology acceptance model (STAM). Ergonomics. 2014;57(5):635-652. doi:10.1080/00140139.2014.895855.

3. Sainimnuan S, Preedachitkul R, Petchthai P, Paokantarakorn Y, Siriussawakul A, Srinonprasert V. Low Prevalence of Adequate eHealth Literacy and Willingness to Use Telemedicine Among Older Adults: Cross-Sectional Study From a Middle-Income Country. J Med Internet Res. 2025;27:e65380. Published 2025 Jul 1. doi:10.2196/65380

4. Georgsson M, Odzakovic E, Björk M, et al. Validation of the eHealth Literacy Scale Instrument in a Restless Legs Syndrome Population: Classical Test Theory and Rasch Analysis Study. J Med Internet Res. 2025;27:e68474. Published 2025 Sep 10. doi:10.2196/68474

5. Wang K, Dong Y, Li ZJ, Cao J, Chang C, Ji Y. Utilization of internet healthcare services and associated factors among older adults in China. Medicine and Society. 2024;37(5):1-9.[Chinese] [doi:10.13723/j.yxysh.2024.05.001]

6. Bauer JM, Brand T, Zeeb H. Pre-migration socioeconomic status and post-migration health satisfaction among Syrian refugees in Germany: A cross-sectional analysis. PLoS Med. 2020;17(3):e1003093. Published 2020 Mar 31. doi:10.1371/journal.pmed.1003093

7. Zhang X, Han X, Dang Y, Meng F, Guo X, Lin J. User acceptance of mobile health services from users' perspectives: The role of self-efficacy and response-efficacy in technology acceptance. Inform Health Soc Care. 2017;42(2):194-206. doi:10.1080/17538157.2016.1200053

**Figure S2. Structure Diagram of Elderly Internet Use Ecological Model**


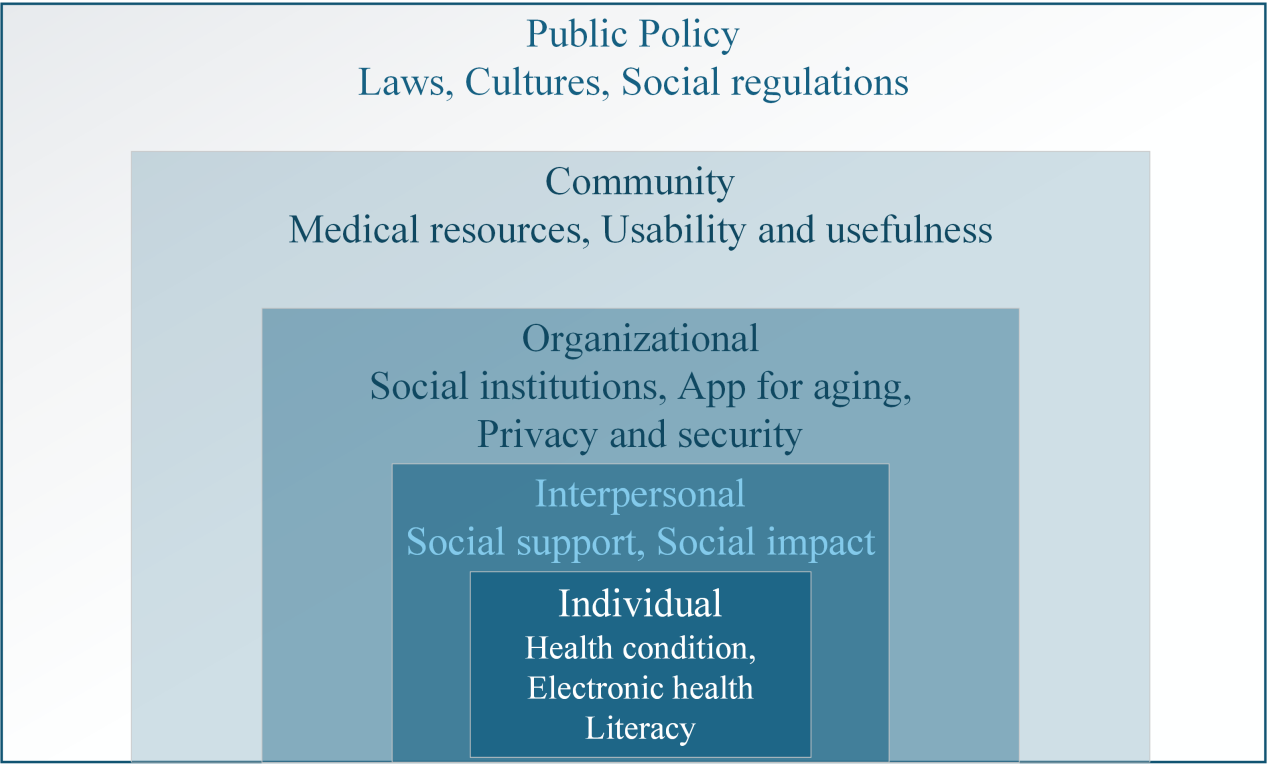


**Table S3. Internet Medical Service Utilization**

|  | Variable | Proportion |
| --- | --- | --- |
| 1 | Do you use online appointment registration? | 772（42.2%） |
| 2 | Do you use online doctor consultations? | 271（14.8%） |
| 3 | Do you schedule a checkup or lab test online? | 343（18.8%） |
| 4 | Do you check your test results online? | 410（22.4%） |
| 5 | Do you use online fee payment? | 543（29.7%） |
| 6 | Do you use online psychological counseling? | 33（1.8%） |
| 7 | Do you use an online medication guide? | 44（2.4%） |
| 8 | Do you use online vaccination appointments? | 109（6.0%） |
| 9 | Have you made an online appointment for in-home care? | 28（1.5%） |
| 10 | Do you use online appointments for medication delivery? | 39（2.1%） |

**Table S4. Comparison Table of AIC, BIC, aBIC, and Likelihood Ratio Test Results for Different Clustering Models**

| Model | AIC | BIC | aBIC | LMR LR  p-value | ALMR LR  p-value | BLRT  p-value |
| --- | --- | --- | --- | --- | --- | --- |
| 1C | 14753.538 | 14814.159 | 14806.546 | <.0001 | - | - |
| 2C | 10086.541 | 10213.293 | 10197.376 | <.0001 | <.0001 | <.0001 |
| 3C | 9071.722 | 9264.606 | 9240.384 | <.0001 | <.0001 | <.0001 |
| 4C | 9054.311 | 9313.327 | 9280.801 | <.0001 | <.0001 | <.0001 |
| 5C | 8556.472 | 8987.752 | 8938.616 | <.0001 | <.0001 | <.0001 |
| 6C | 8562.904 | 9020.315 | 8962.874 | <.0001 | <.0001 | <.0001 |

Note: aBIC: Adjusted BIC; AIC: Akaike Information Criterion:ALMR LR: Adjusted LMR LR; BIC: Bayesian Information Criterion; BLRT: bootstrap likelihood ratio test;LMR LR;L o-Mendell-Rubin likelihood ratio.

**Figure S3.** **Radar Chart of Internet Usage with Different Clustering Models**
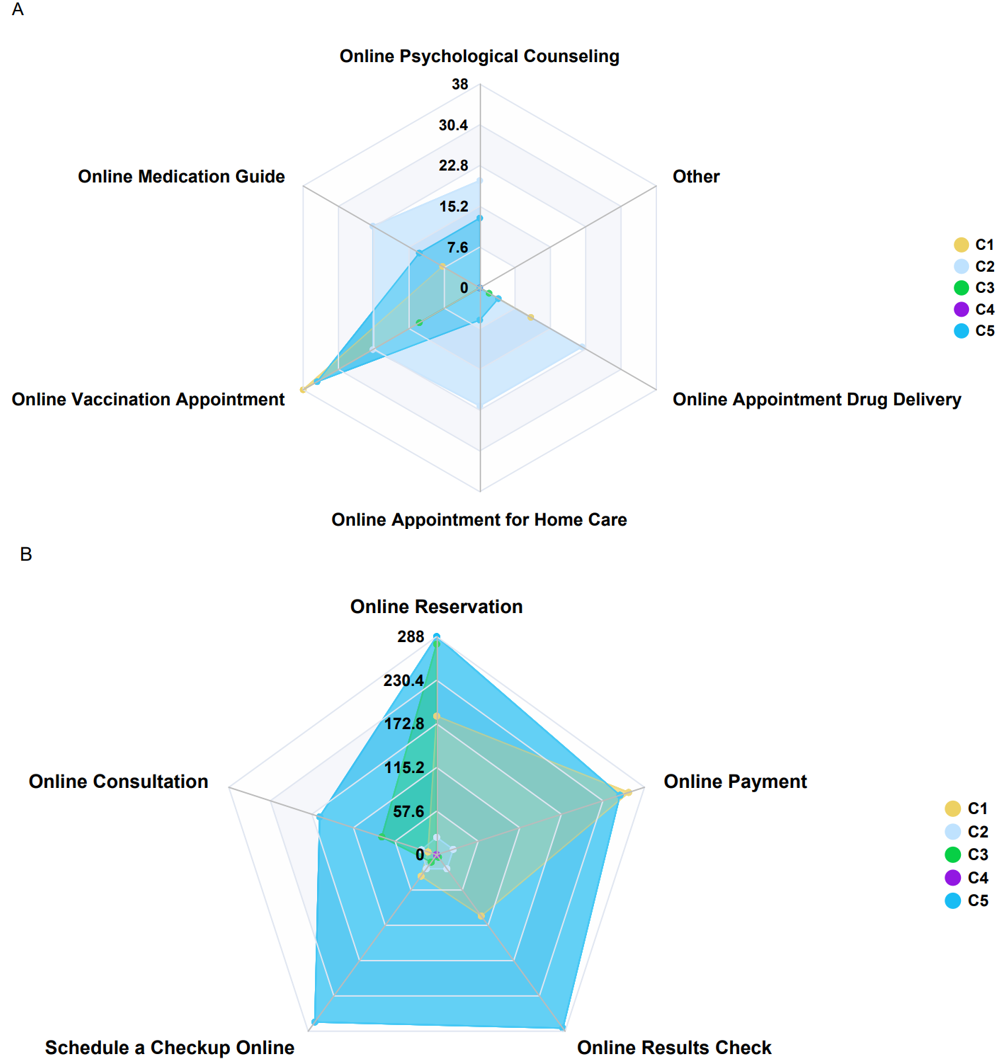


**Figure S4. Sankey Diagram of Internet Usage with Different Clustering Models(A: Technical Acceptance; B: Health Status Score; C: Total Chronic Diseases; D: Social Support)**


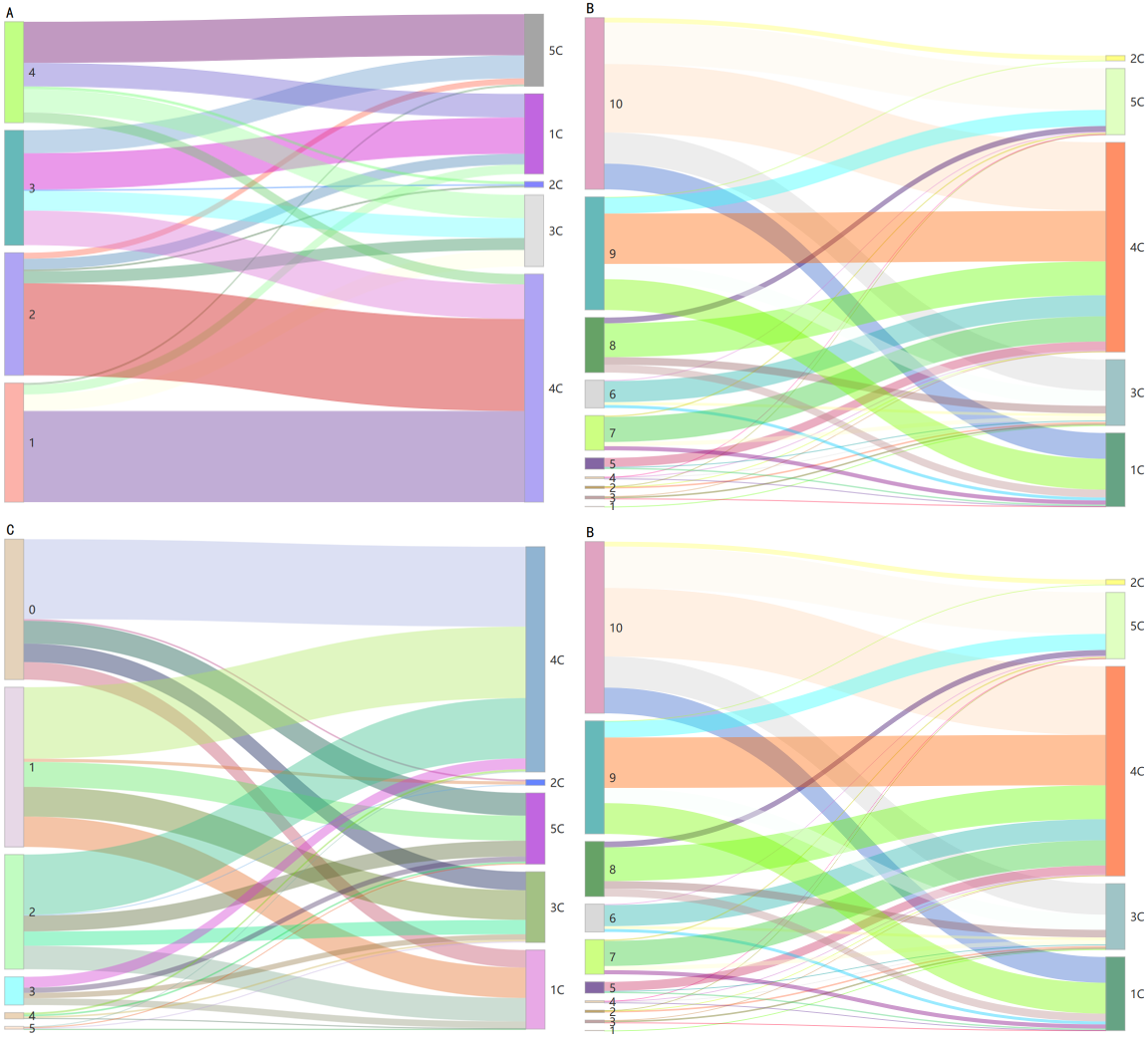


On the right side, numbers 1–5 represent different classification categories (e.g., class 1, class 2, etc.), illustrating the flow and distribution relationships between various categories for each clustering model.A certain color - coded block on the left is significantly wider, it implies that this technical acceptance level has a dominant proportion in the corresponding classification category it flows into

**Table S5. Comparison Table of Sensitivity, Specificity, Accuracy and Other Performance Indicators between Test Set and Training Set**

|  | **Sensitivity** | **Specificity** | **Precision** | **Accuracy** | **F1 score** | **False positive rate** |
| --- | --- | --- | --- | --- | --- | --- |
| Test set | .8644 | .9017 | .9049 | .8823 | .8842 | .0982 |
| Training set | .8863 | .8565 | .8484 | .8707 | .8669 | .1434 |
